# Sir C. Pegge, on the Origin of the Vaccine Disease

**Published:** 1800-11

**Authors:** Christ. Pegge

**Affiliations:** Oxford


VOL. IV.] NOVEMBER, 1800. [NO. XXI.

### *To the Editors of the Medical and Physical Journal.*

GENTLEMEN,

I Shall feel myself obliged to you to insert the inclosed letter to Dr. JENNER in your interesting publication, the Medical and Physical Journal. I am,

GENTLEMEN,

Your obedient servant,

*Oxford, Oct.* 16, 1800. CHRIST. PEGGE.

---

### *To* DR. JENNER.

DEAR SIR,

I have the honour of transmitting to you a series of facts respecting the Cow-Pox, which appear to me to be very interesting; and I flatter myself will be the more so to you, as they tend to establish your own opinion as to the origin of that disease.

They have been communicated to me as they arose by a friend of mine, Mr. Lupton of Thame, a surgeon of long experience, and I do not hesitate to add, a man of the greatest respectability in every point of view.

The attention of Mr. Lupton was first drawn to the present subject in March last, when the son of Mr. Way, farmer, of Ichford, applied to him on account of a complaint in his hand, attended with ulcerations very much resembling the Cow-Pox. There was evidently a very great derangement of the system, and the symptoms plainly indicated an absorption of morbid matter, as the case was also attended with considerable swelling of the hand and arm, an enlargement of the axillary glands, rigors, pain in the hand and back, together with a greatly increased quickness of the circulation.

He could only account for these complaints from his having washed the ulcerated heels of a horse, having had no previous communication with the cows.

Thefe circumftances led Mr. Lupton to conceive that there might be a difeafe incident to the horfe analagous to the Cow-Pox, and communicable to the cow; and upon repeated enquiry he was fatisfied that it was not the common greafe to which horfes are liable, that had produced the above effects.

Mr. Lupton was fo good as to communicate this information to me at the time, treating it as a matter of curiofity, rather than of ferious inveftigation, and I heard no more from him on this fubject till the 8th of April, when I received the following letter, which I give you in his own words.

Dear Sir,

Since my laft letter refpecting Mr. Way's fon, I have had another cafe of infection, communicated to the human fubject, owing to matter abforbed from the ulcerated heels of a horfe. The perfon is Richard Hunt, a fervant of Mr. Randolph, of Thame Park Farm, whofe firft fymptoms were ftiffnefs and uneafinefs of the arm, fwelling of the axillary glands, fucceeded by puftules on the hand, and a very painful fuppuration of the middle finger, which had that blue appearance defcribed by Dr. Jenner, as indicating the true vaccine difeafe; thefe were accompanied with rigors frequently recurring, attended with great heat, anxiety, giddinefs, pain in the head and back, ficknefs, and vomiting.

Such were the appearances when I firft faw him, which was on Sunday, March 30. On the 31ft, he had a very bad night, and had been flightly delirious, the other arm growing ftiff and painful. April 1ft, was much better in every refpect, except the painful ftate of the finger, and the inflammation of the hand and arm: the courfe of the lymphatics were at this time beautifully marked with ftreaks of a vivid red colour extending from the wrift to the axilla. April 2d, continued better. April 3d, had a bad night from the pain of the finger; a puncture was now made, and about two tea-fpoons full of a dark brown coloured fluid were difcharged. April 4th, the cuticle was now removed, and difcovered a fhining red ulcerated furface, in the middle of which was a fpot of a floughy appearance, of the fize of a filver penny; this was covered with the red nitrate of quickfilver: the inflammation, pain, and fwelling of the hand and arm were now confiderably abated, and in other refpects he was much relieved. April 6th, the finger much better; the puftules of the hand had a dark coloured depreffion in the centre, furrounded with an elevated margin of matter. From this time he had no complaint. It muft be particularly remarked that this man has not milked any cow fince laft Michaelmas.

*Thame, April 8, 1800.*

On

On the 9th of April, a ſecond ſervant of Mr. Randolph, John Watſon, applied to Mr. Lupton, with ſymptoms ſimilar to thoſe of Richard Hunt, in conſequence of having aſſiſted him in dreſſing the heels of the horſe; Watſon's office and employment in the farm at this time was that of milking the cows.

Previous, however, to the appearance of ulceration upon the man's hands, the cows had been infected more than a week, and there can be no doubt but that the cows had received their infection from the horſe, through the medium of this ſervant. Whether the hands of this man were ulcerated from the immediate effect of the matter received from the heels of the horſe, as on that occaſion he was aſſiſtant only; or immediately from its modification in the teats of the cow, may admit of doubt. But from the fulleſt inveſtigation, I am perſuaded there can be no doubt as to the fact of the matter having been conveyed from the horſe's heels to the cows by this laſt mentioned ſervant.

Such was the progreſs of this hiſtory previous to the 18th of May, when happening to be at Thame, Mr. Lupton informed me that a third ſervant of Mr. Randolph (Leonard Paling) was affected in a manner ſimilar to that in which the two former had been attacked. In conſequence of this I had the curioſity to ſend for him; and on examination we were thoroughly convinced that he had received the infection ſolely from the cows, as he had never aſſiſted in dreſſing the heels of the horſe.

I found the appearances on his hand exactly correſponding with thoſe already deſcribed; I obſerved one of the axillary glands ſtill enlarged and very tender; and from his own ſtatement his whole ſyſtem had been very much diſturbed, evidently the effect of the ulceration on his hand.

Thus, dear ſir, we have ſeen a complete ſeries of facts relative to the progreſs of this very important diſeaſe as affecting the human frame, eſtabliſhing its origin in a diſorder of the horſe's heels, by farriers termed a ſcratchy heel, and conſidered as widely different from common greaſe.

The firſt ſervant was infected from the horſe, having never milked the cows; the ſecond carried the infection from the horſe to the cows; and the third received it ſolely from the cows.

I have only to add that from the laſt ſervant, Leonard Paling, Mr. Lupton inoculated ſeveral children.

Some of theſe I ſaw on the 8th day after inoculation, with the moſt decided appearance of true Cow-Pox upon them. This appearance, I could not miſtake after having witneſſed ſo many inſtances of it at our friend's, Mr. Fermor of Tuſmore, whoſe

 benevolent benevolent and disinterested exertions have contributed so largely to the stock of facts in support of a discovery, which, if the powerful argument of induction may be allowed, bids fair to be of the greatest benefit to mankind. It is proper to add, that in all these children the disease terminated as usual, favourably.

One circumstance was remarkable in all the three servants above mentioned, viz. that before any absolute ulceration or even sensation of pain took place on their hands, they had been previously affected by swellings in the axilla, and other symptoms denoting constitutional disease; and the second servant (Watson) assured me repeatedly, that though both his hands had been ulcerated, a swelling first appeared in the axilla of the left arm, was followed by sickness, head-ache, &c. &c.; after which the ulceration commenced in the palm of the right hand between the fore finger and thumb.

As not one of the persons above mentioned have had the Small-Pox, it is Mr. Lupton's intention to inoculate them as soon as he can find a suitable opportunity; and when that occurs you shall be informed of the result. I have the honour to be,

DEAR SIR,

Your very sincere humble servant,

*Oxford, Oct.* 3, 1800. CHRIST. PEGGE.

